# Weighted gene coexpression network and experimental analyses identify lncRNA SPRR2C as a regulator of the IL-22-stimulated HaCaT cell phenotype through the miR-330/STAT1/S100A7 axis

**DOI:** 10.1038/s41419-020-03305-z

**Published:** 2021-01-15

**Authors:** Meijunzi Luo, Pan Huang, Yi Pan, Zhu Zhu, Rong Zhou, Zhibo Yang, Chang Wang

**Affiliations:** 1 Department of Dermatology, the Second Affiliated Hospital, The Domestic First-class Discipline Construction Project of Chinese Medicine of Hunan University of Chinese Medicine, Changsha, 410005 Hunan China; 2 The Second Clinical College of Chinese Medicine, Hunan University of Chinese Medicine, Changsha, 410005 Hunan China

**Keywords:** Cells, Diseases

## Abstract

Psoriasis is a chronic inflammatory disease of the skin with highly complex pathogenesis. In this study, we identified lncRNA SPRR2C (small proline-rich protein 2C) as a hub gene with a critical effect on the pathogenesis of psoriasis and response to treatment using both weighted gene coexpression network analysis (WGCNA) and differential expression analysis. SPRR2C expression was significantly upregulated in both psoriatic lesion samples and HaCaT cell lines in response to IL-22 treatment. After SPRR2C knockdown, IL-22-induced suppression of HaCaT proliferation, changes in the KRT5/14/1/10 protein levels, and suppression of the IL-1β, IL-6, and TNF-α mRNA levels were dramatically reversed. In the coexpression network with SPRR2C based on GSE114286, miR-330 was significantly negatively correlated with SPRR2C, while STAT1 and S100A7 were positively correlated with SPRR2C. By binding to miR-330, SPRR2C competed with STAT1 and S100A7 to counteract miR-330-mediated suppression of STAT1 and S100A7. MiR-330 overexpression also reversed the IL-22-induced changes in HaCaT cell lines; in response to IL-22 treatment, miR-330 inhibition significantly attenuated the effects of SPRR2C knockdown. STAT1 and S100A7 expression was significantly upregulated in psoriatic lesion samples. The expression of miR-330 had a negative correlation with the expression of SPRR2C, while the expression of SPRR2C had a positive correlation with the expression of STAT1 and S100A7. Thus, SPRR2C modulates the IL-22-stimulated HaCaT cell phenotype through the miR-330/STAT1/S100A7 axis. WGCNA might uncover additional biological pathways that are crucial in the pathogenesis and response to the treatment of psoriasis.

## Introduction

Psoriasis is a chronic inflammatory disease of the skin affecting >2% of the population worldwide and is characterized by sharply demarcated, erythematous, scaling plaques that typically affect the elbows, knees, scalp, and trunk. Patients with psoriasis have a high risk of developing psoriatic arthritis, metabolic syndrome, and cardiovascular disease^1^. In addition to conventional therapies, the management of psoriasis includes a new generation of inhibitors that target immune response factors, such as TNF-α, IL-12-23, IL-17, JAK, and PDE4^2–6^; however, the treatment efficiency remains unsatisfactory due to the complicated pathogenesis of the disease.

In recent years, high-throughput sequencing technologies such as gene chips and RNA-seq have emerged, allowing the generation of large-scale omics data^7–10^, and the number of differentially expressed genes (DEGs) found between psoriatic lesions and normal control skin is continuously increasing^9^. However, focusing on a single or a few genes from a local perspective is insufficient to explain the highly complex pathogenesis of psoriasis^11–13^. Based on the regulatory network as a whole, some genes in lesion tissues are abnormally expressed and are closely related to each other. Their expression levels may exert a critical effect on the initiation and progression of psoriasis. Such genes at the hub of the regulatory network are called hub genes^14^. Investigating the hub genes involved in the occurrence and development of psoriasis based on online datasets might provide a novel perspective for understanding the highly complex pathogenesis of psoriasis. In the field of bioinformatics, the application of network analysis is becoming increasingly mature. Among these techniques, as a new method with potential in systems biology analysis, weighted gene coexpression network analysis (WGCNA) has advantages in describing and analyzing molecular mechanisms and network relationships^15^. For the identification of a biomarker, screening based on WGCNA achieved a better validation rate than screening based on differential expression analysis. Since its development, WGCNA has been successfully used in multiple biological environments and has achieved a series of research results in psoriasis^9,16–19^.

Long noncoding RNAs (lncRNAs) are a type of RNA defined as transcripts with lengths exceeding 200 nucleotides that are not translated into protein^20^. LncRNAs exert critical effects on the regulation of immune-mediated inflammatory diseases and autoimmune diseases^21,22^. In many immune disorders, such as psoriasis, lncRNA dysregulation has been researched^16,23,24^, suggesting that lncRNAs have important roles in regulating immune-mediated disorders and are involved in psoriatic pathogenesis by modulating protein-coding gene expression or by changing the structure of chromatin^25,26^. WGCNA has been applied in the identification of psoriasis-related coding gene networks^9,27^ but has not been widely applied in the identification of coding gene and lncRNA networks. Thus, we employed WGCNA to coding genes and lncRNAs using online RNA-seq and microarray expression profiles to identify new coding gene and lncRNA networks related to psoriatic pathogenesis and response to treatment.

Regarding further downstream molecular mechanisms, lncRNAs act on *cis*- and *trans*-regulated target genes, thus exerting critical functional effects in epigenetic, transcriptional, or post-transcriptional modulation^28,29^. LncRNAs form extensive networks of ribonucleoprotein (RNP) complexes^28^. In recent decades, it has been widely accepted that lncRNAs competitively bind to microRNAs (miRNAs), another type of short noncoding RNAs, to relieve miRNA-induced repression of miRNA target genes^30,31^. Therefore, we continued to investigate miRNAs that might be related to identified novel networks of coding genes and lncRNAs. The in vitro effects of the identified lncRNAs and miRNAs were examined alone and combined, the predicted binding of miRNAs to lncRNAs and coding genes was validated, and the expression of related factors was examined in tissue samples. In summary, we report the identification of new coding gene, lncRNA, and miRNA networks related to psoriatic pathogenesis and response to treatment and suggest that WGCNA reveals additional biological pathways.

## Materials and methods

### Clinical psoriatic lesion and nonlesion tissue samples

A total of 12 psoriatic lesion tissues and 12 nonlesion tissues were collected from patients with psoriasis who underwent treatment at the Second Affiliated Hospital of the Hunan University of Chinese Medicine with signed informed consent and the approval of the Research Ethics Committee of The Second Affiliated Hospital of Hunan University of Chinese Medicine. None of the patients has been treated with systemic immunosuppressive medications for 4 weeks before the participation in the study. All the patients have signed informed consent. The tissue samples were stored at formalin or at −80 °C immediately after sampling until further experiments. The severity of psoriasis was monitored, and the Psoriasis Area Severity Index (PASI) was determined^32^.

### Histopathological analyses by hematoxylin and eosin (H&E) staining

Tissue samples were fixed in 10% formalin solution, embedded in paraffin, and processed following the standard procedure^33^. The samples were then cut into 5-μm-thick slices and stained with H&E for histopathological analyses.

### Immunohistochemistry (IHC) staining

Sample slides were deparaffinized, rehydrated, and incubated with proteinase K for 15 min. Nonspecific binding was blocked using 3% BSA blocking solution. Then, the slices were incubated with anti-S100A7 and anti-STAT1 primary antibodies overnight at 4 °C. The following steps were performed using the PolyExcel HRP/DAB Detection System kit (PathnSitu Biotechnologies, Livermore, CA, USA) according to the manufacturer’s instructions. Signal visualization was generated using DAB (3,3′-diaminobenzidine tetrachloride), and the sections were counterstained with hematoxylin for IHC analysis.

### Immunoblotting

The protein levels of KRT5, KRT14, KRT1, KRT10, STAT1, and S100A7 were examined by immunoblotting following the methods described before^34^ with primary antibodies against KRT5 (ab52635; Abcam, Cambridge, MA, USA), KRT14 (ab7800, Abcam), KRT1 (ab93652, Abcam), KRT10 (ab76318, Abcam), STAT1 (ab109320, Abcam), and S100A7 (ab13680, Abcam) and the HRP-conjugated secondary antibody. Signals were visualized using enhanced chemiluminescent (ECL) substrates (Millipore, MA, USA) with normalization to GAPDH.

### RNA extraction and real-time PCR analysis

Total RNA was extracted from cultured cells using TRIzol reagent (Invitrogen). The miRNA and mRNA levels were measured using a SYBR Green qPCR assay (TaKaRa, Dalian, China) following the methods described before^34^. The expression of GAPDH (reference for mRNA determination) or RNU6B (reference for miRNA determination) served as an endogenous control. The sequences of the PCR primers are presented in Supplementary Table S1. The 2^−ΔCT^ method was applied for data processing.

### Cell line, cell treatment, and cell transfection

Human immortalized keratinocytes, HaCaT cells, were obtained from ProCell (CL-0090, Wuhan, China) and cultured in MEM medium (PM150410, ProCell) supplemented with 15% FBS (164210-500, ProCell) and 1% P/S (PB180120, ProCell). The cells were cultured at 37 °C in 5% CO_2_.

For recombinant human IL-22 (Sangon Biological Engineering Technology Company, Shanghai, China) treatment, HaCaT cells were starved in serum-free DMEM for 24 h and then treated with IL-22 (100 ng/ml) in serum-free DMEM for another 24 h or not treated.

SPRR2C (small proline-rich protein 2C) silencing was generated by the transfection of si-SPRR2C1/2/3 (GenePharma, Shanghai, China). MiR-330 overexpression or inhibition was conducted by the transfection of miR-330-5p or antagomir-330-5p (GenePharma). The primer sequences of si-SPRR2C1/2/3 or mir-330-5p are listed in Supplementary Table S1. The transfection was performed using Lipofectamine 3000 (Invitrogen, Carlsbad, CA, USA).

### Cell viability determination by MTT assays

The cell viability of target cells (transfected or treated) was determined using the MTT assay as described previously^34^. DMSO was added after the supernatant was discarded to dissolve the formazan. OD values were measured at 490 nm. The viability of cells in different groups was calculated taking the viability of the nontreated cells (control) as 100%.

### DNA synthesis determined by EdU assays

DNA synthesis was determined based on the method of incorporating thymidine analog 5-ethoxy 2-deoxyuridine (EdU) into genomic DNA by using the Click-IT EdU Alexa Fluor 488 kit following the methods described before^35,36^. Apollo staining and DAPI staining were performed, and EdU-positive cells were observed with a fluorescence microscope. The incorporation rate of EdU was calculated as the ratio of EdU-positive cells (green cells) to total DAPI-positive cells (blue cells).

### LncRNA–miRNA–mRNA correlations verified by luciferase reporter assays

To verify the predicted binding of lncRNA SPRR2C, miR-330, STAT1, and S100A7, we performed a luciferase reporter assay by constructing wild-type and mutant-type SPRR2C or STAT1 3′UTR and S100A7 3′UTR reporter vectors. For SPRR2C-miR-330-STAT1, wild-type vectors contained the wild-type SPRR2C fragment or STAT1 3′UTR possessing the predicted miR-361-binding site; mutant-type vectors contained the mutated SPRR2C fragment or STAT1 3′UTR possessing several mutations in the predicted miR-361-binding site. These vectors were cotransfected with miR-361 mimics or inhibitors. For SPRR2C-miR-361, wild-type vectors contained the wild-type SPRR2C fragment or S100A7 3′UTR possessing the predicted miR-330-binding site; mutant-type vectors contained the mutated SPRR2C fragment or S100A7 3′UTR possessing several mutations in the predicted miR-361 binding site. These vectors were cotransfected with miR-330 mimics or inhibitors. Then, the changes in luciferase activity were monitored using the Dual-Luciferase Reporter Assay System (Promega, Madison, MI, USA) 48 h after transfection. The primer sequences of wild-type and mutant-type SPRR2C, STAT1, and S100A7 are listed in Supplementary Table S1. Renilla luciferase activity was normalized to firefly luciferase activity for each transfected well.

### Statistical analysis

Data from at least three independent experiments were processed using SPSS17.0 (IBM, Armonk, NY, USA) and are presented as the mean ± S.D. Student’s *t* test was used for statistical comparison between means where applicable. Differences among more than two groups in the above assays were estimated using one-way ANOVA. *P < 0.05; **P < 0.01.

## Results

### Screening for coding genes and lncRNAs associated with the pathogenesis of psoriasis and response to therapy using WGCNA (weighted gene coexpression network analysis) and differential expression analysis

For WGCNA, we chose the GSE30999 microarray expression profile containing 22188 differentially expressed genes in total from 170 cases of psoriatic skin lesions and normal control tissue samples. The genes without significant differential expression were filtered through Multi Explorer Viel software, and the top 5000 differentially expressed genes were retained for the following analysis. Hierarchical clustering was used to obtain a sample dendrogram and trait heatmap of the psoriatic samples and normal control tissue samples (Supplementary Fig. S1A); the genes in the psoriatic lesional and normal control tissue samples had good clustering. Outliers were removed by pruning. The cluster in Supplementary Fig. S1B shows 11 gene coexpression modules formed by these 5000 differentially expressed genes. Different gene coexpression modules are represented in red, green-yellow, blue, yellow, pink, green, turquoise, red-brown, purple, black, and magenta. Gray represents genes not successfully clustered. Next, we selected the appropriate adjacency matrix weight parameter β to ensure a scale-free distribution as much as possible. The parameter β used a value from 1 to 20. A linear model of the logarithm of the connectivity of a node log (i) and the logarithm of the probability of the node’s appearance log (p (i)) was established. The parameter β was the square of the coefficient R. According to the candidate parameter β, the detected network parameters are shown in Supplementary Fig. S1C. When β was 6, the evaluation parameters of the scale-free network could be close to 0.9, so β = 6 had a good discrimination degree and was used for further analysis. The network topology overlaps or Topological Overlap Matrix (TOM) was calculated using β = 6, and the genetic system cluster tree was obtained using the hierarchical clustering method (Supplementary Fig. S1D). Through WGCNA, we identified highly related gene modules in psoriatic pathological features, that is, genes represented by coexpression modules are closely related in function. The correlation between the 11 gene coexpression modules is shown in Supplementary Fig. S1E. It can be seen that the turquoise, green, and red-brown modules have better clustering, while the purple, black, and magenta modules show clustering, and the relationship between yellow, pink, and blue is close. Correlation analysis was performed between the module and clinical features (psoriatic pathological lesions and normal tissues); the turquoise module had a very significant positive correlation with clinical features of psoriasis (*r* = 0.92, *P* = 3e-88) and had a strong negative correlation with normal control tissues (Supplementary Fig. S1F). Thus, we performed GO analysis on the turquoise module. The results showed that genes in the turquoise module are related to skin defense and inflammation. The main related processes include the production of inflammatory factors (inflammation), endopeptidase activity (bactericidal), and bacterial lipopolysaccharide response (immunity), which are all related to psoriatic pathogenesis. In the turquoise module, a total of 81 lncRNAs were found. The information of the datasets we used in this study is shown in Supplementary Table S2.

Next, two RNA-seq datasets and six microarray expression profiles were assessed for differentially expressed lncRNAs between normal control and psoriatic lesional tissue samples (Supplementary Fig. S2A). As shown in Supplementary Fig. S2B–H, a total of four lncRNAs, SPRR2C, TMEM254-AS1, EPB41L4A-AS1, and SH3PXD2A-AS1, were significantly differentially expressed between the nonlesion and lesion tissues and/or before and after treatment in the lesion tissues. Among them, TMEM254-AS1 and EPB41L4A-AS1 were significantly downregulated in the lesion tissues, while SH3PXD2A-AS1 and SPRR2C were significantly upregulated (Supplementary Fig. S2B–H); SPRR2C was one of the most upregulated genes in the lesion tissues (Supplementary Fig. S2I).

Finally, after cross-check analysis, SPRR2C was selected by both WGCNA and differential expression analyses (Supplementary Fig. S3A). More importantly, compared to that in the lesion tissues without treatment, the expression of SPRR2C was substantially inhibited in the psoriatic lesion samples under treatment at different time points (Supplementary Fig. S3B–D). Compared to that in the cases with relapse, SPRR2C expression was significantly downregulated in the nonrelapse cases (Supplementary Fig. S3E). SPRR2C expression was significantly correlated with PASI scores (Supplementary Fig. S3F). These data indicate that lncRNA SPRR2C might be a hub gene in psoriatic pathogenesis and response to treatment.

### Expression of lncRNA SPRR2C in psoriatic tissue samples

**Fig. 1:**
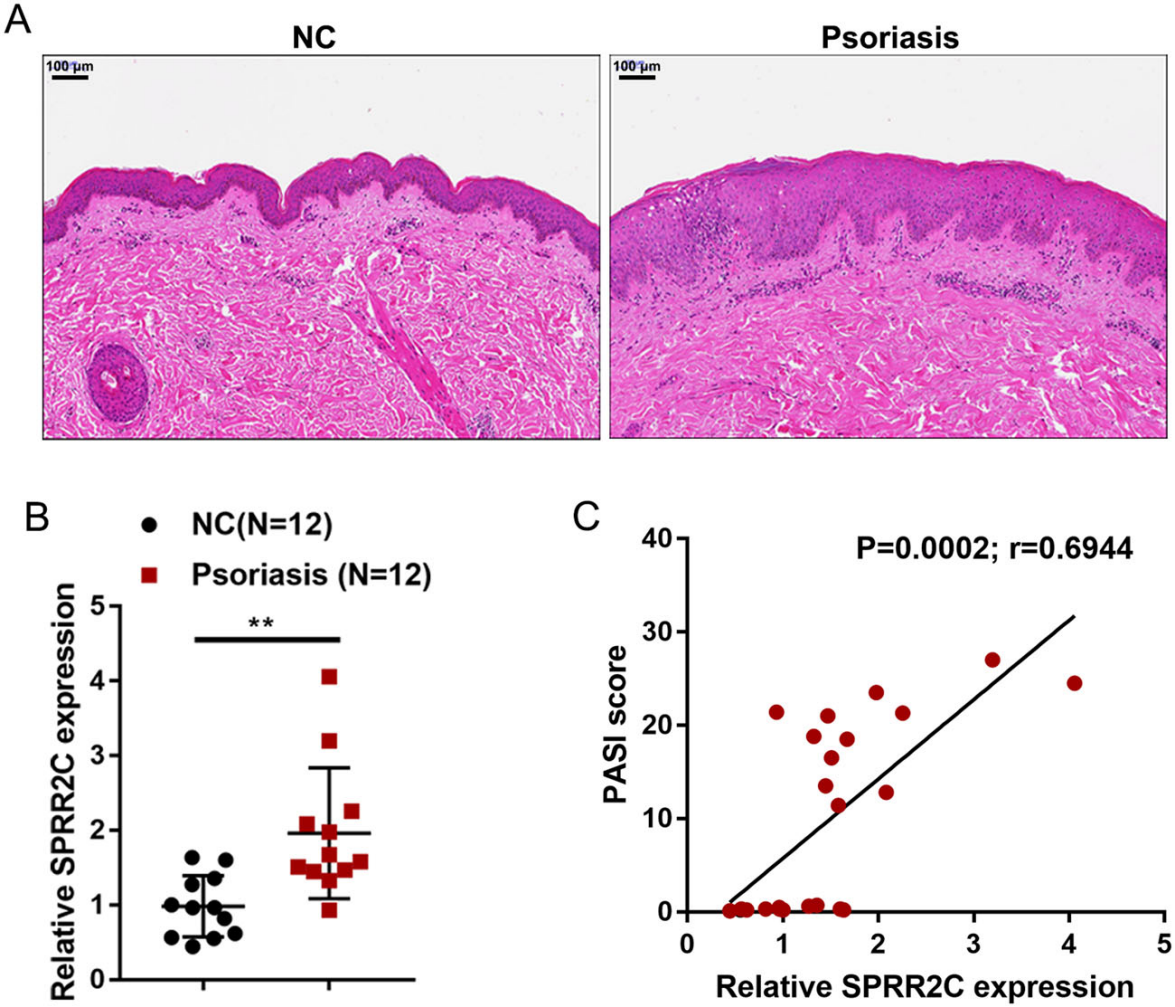
Expression of long noncoding RNA (lncRNA) SPRR2C (small proline-rich protein 2C) in psoriatic tissue samples. **A** The histopathological features of the psoriatic lesion and nonlesion tissues examined by hematoxylin and eosin (H&E) staining. **B** The expression of SPRR2C was determined in 12 psoriatic lesions and 12 nonlesion tissues by real-time PCR. **C** The correlation of SPRR2C expression and Psoriasis Area and Severity Index (PASI) scores in clinical cases was determined using Pearson’s correlation analysis.

To validate the expression of SPRR2C in psoriatic lesions, we first collected clinical samples and examined the histopathological features of the psoriatic lesions and nonlesion tissues by H&E staining. As shown in Fig. 1A, hyperkeratosis with parakeratosis, dilated dermal blood vessels, and inflammatory infiltration could be observed in psoriatic lesions. In the psoriatic lesion tissues, the expression of SPRR2C was significantly upregulated compared with that in the normal control tissues (Fig. 1B). More importantly, SPRR2C expression in the tissue samples was positively correlated with the Psoriasis Area and Severity Index (PASI) scores in clinical cases (Fig. 1C). These data further confirm the WGCNA and microarray expression profile analyses results and suggest that the abnormal upregulation of SPRR2C in psoriasis might contribute to the pathogenesis of this disease.

### Effects of SPRR2C on IL-22-induced human immortalized keratinocytes

**Fig. 2:**
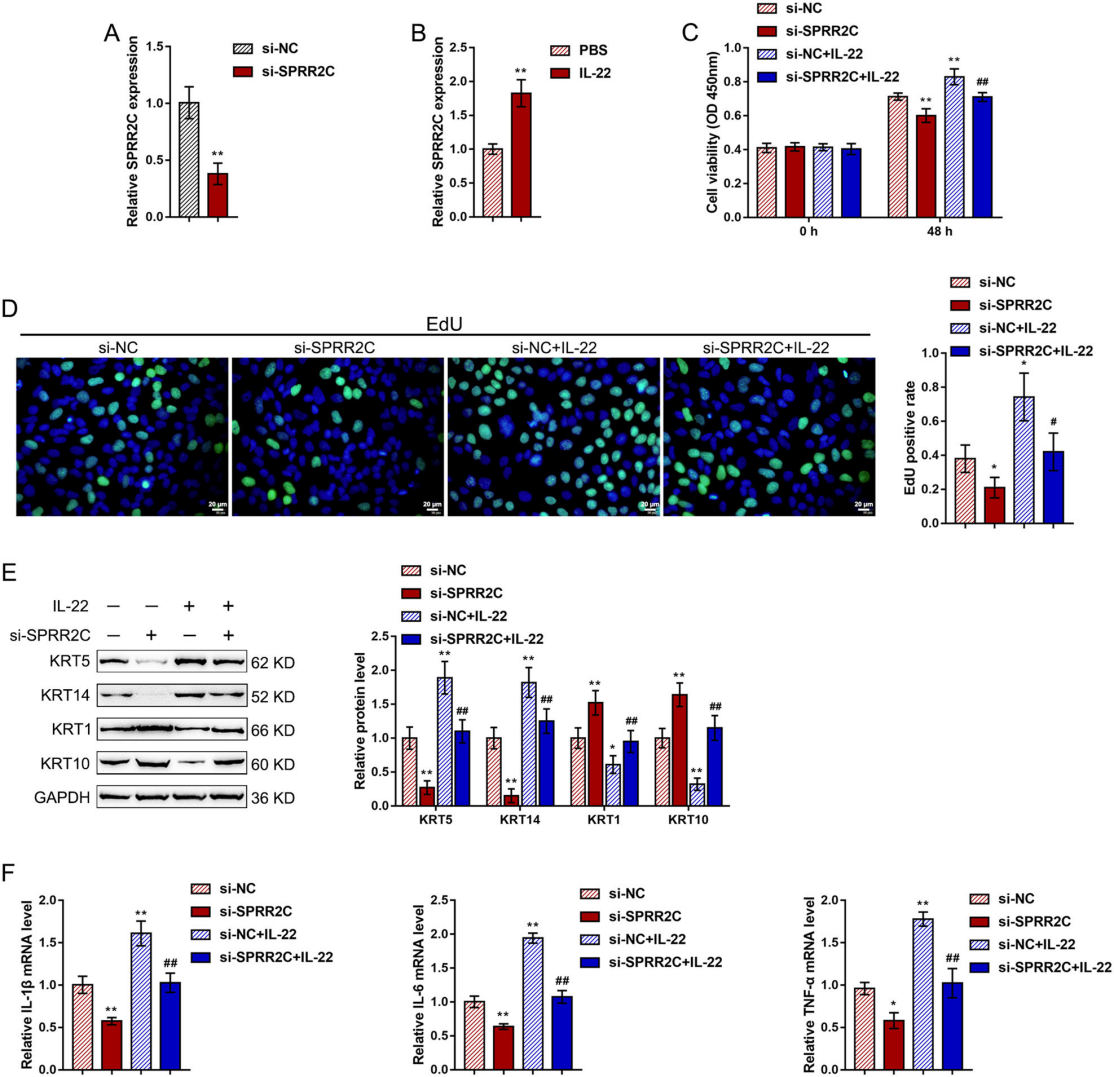
Effects of SPRR2C on IL-22-induced human immortalized keratinocytes. **A** SPRR2C knockdown was conducted in human immortalized keratinocytes (HaCaT cells) by the transfection of si-SPRR2C1/2/3; the transfection efficiency was confirmed by real-time PCR. Si-SPRR2C1 was selected for further experiments because of its high transfection efficiency. **B** HaCaT cells were starved in serum-free DMEM for 24 h, treated with IL-22 (100 ng/ml) in serum-free DMEM for another 24 h or not treated, and then examined for the expression of SPRR2C. Next, the HaCaT cells were transfected with si-SPRR2C in the presence or absence of IL-22 stimulation and examined for (**C**) cell viability by MTT assays; **D** DNA synthesis by EdU assays; **E** the protein levels of KRT5/14/1/10 by immunoblotting; **F** and the mRNA expression levels of IL-1β, IL-6, and TNF-α by real-time PCR. *P < 0.05, **P < 0.01, compared to the control group; ^#^*P* < 0.05, ^##^*P* < 0.01, compared to the si-NC+IL-22 group.

To investigate the specific effects of SPRR2C, we stimulated human immortalized keratinocytes (HaCaT cells) with IL-22 (100 ng/ml) for 24 h to mimic the in vitro pathological changes in psoriasis, conducted SPRR2C knockdown in HaCaT cells, and then examined related indexes. SPRR2C knockdown was conducted by the transfection of si-SPRR2C1/2/3; we performed real-time PCR to verify the transfection efficiency and selected si-SPRR2C1 for further experiments because it exhibited the highest transfection efficiency (Fig. 2A). Upon IL-22 stimulation, SPRR2C expression was significantly upregulated (Fig. 2B). In addition, IL-22 stimulation dramatically enhanced HaCaT cell viability (Fig. 2C) and their ability to synthesize DNA (Fig. 2D), increased K5/14 while decreasing the K1/10 protein levels (Fig. 2E), and upregulated the mRNA levels of inflammatory factors, IL-1β, IL-6, and TNF-α (Fig. 2F); SPRR2C knockdown exerted the opposite effects by inhibiting the cell viability (Fig. 2C) and DNA synthesis (Fig. 2D) of HaCaT cells, decreasing K5/14 while increasing the K1/10 protein levels (Fig. 2E), and downregulating the mRNA expression levels of inflammatory factors, IL-1β, IL-6, and TNF-α (Fig. 2F). Moreover, the effects of IL-22 stimulation on HaCaT cells could be partially reversed by SPRR2C knockdown, suggesting that SPRR2C knockdown could inhibit IL-22-stimulated changes in HaCaT cell lines in vitro.

### Selection of miRNAs related to SPRR2C functions in IL-22-stimulated HaCaT cell lines

**Fig. 3:**
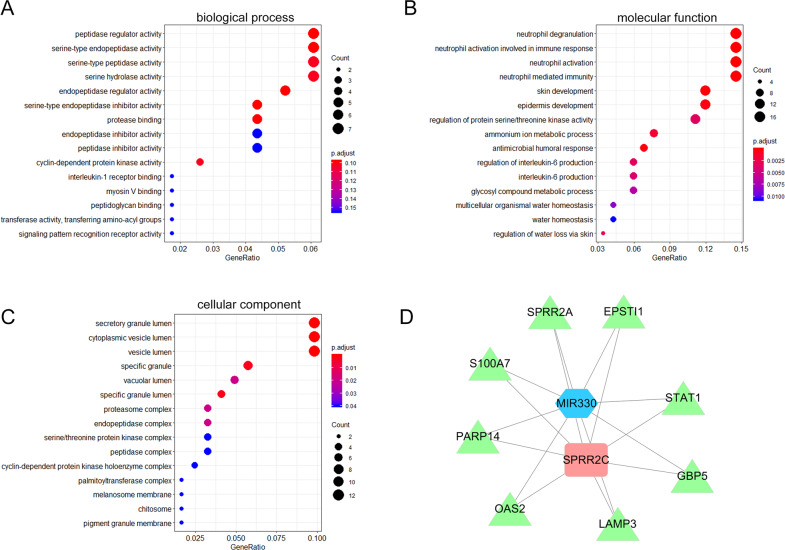
Selection of miRNAs related to SPRR2C functions in IL-22-induced HaCaT cells. The coexpression network of SPRR2C was constructed based on data from GSE114286; coexpressed genes with SPRR2C were applied for Gene Ontology (GO) enrichment analysis. The results of the biological process (BP; **A**), molecular function (MF, **B**), and cellular component (CC, **C**) analyses are shown. **D** Coexpression network of SPRR2C and negative correlation of SPRR2C with MIR-330 based on the data from GSE114286.

The mechanism by which lncRNAs competitively bind to miRNAs to counteract miRNA-mediated repression of miRNA targets has been widely reported^30,31^. Next, we performed a bioinformatics analysis to detect miRNAs that could be associated with SPRR2C function in the pathogenesis of psoriasis. Cases from the RNA-seq dataset GSE114286 were grouped using the median value of SPRR2C expression as the cutoff, and the coexpression network of SPRR2C was constructed; coexpressed genes with SPRR2C were then applied for the Gene Ontology (GO) enrichment analysis. The results showed that genes coexpressed with SPRR2 were significantly enriched in peptidase regulatory activity, serine endopeptidase activity, serine peptidase activity, and serine hydrolase according to biological process (BP, Fig. 3A) analysis; enriched in neutrophil degranulation, neutrophil activation contributing to immune reaction, neutrophil activation, and neutrophil-mediated immunity according to molecular function (MF, Fig. 3B) analysis; and enriched in secretory granule lumen, cytoplasmic vesicle lumen, and vesicle lumen according to cellular component (CC, Fig. 3C) analysis. These results are consistent with earlier GO analysis showing that the genes in the turquoise module obtained in the WGCNA were mainly related to the production of inflammatory factors (inflammation), endopeptidase activity (bactericidal), and bacterial lipopolysaccharide response (immunity), which are all closely related to the pathological state of psoriasis.

In the coexpression network of SPRR2C based on GSE114286, five miRNAs (miR-330, miR-663a, miR-4489, miR-4720, and miR-4725) were markedly negatively correlated with SPRR2C (Supplementary Table S3 and Fig. 3D). In our previous study, we reported that miR-330 targets CTNNB1 to suppress the inducible effects of IL-22 on the proliferation of HaCaT cells and HKC cells and affects the downstream factors CyclinD1 and Axin2^37^; thus, miR-330 was selected for further experiments.

### MiR-330 directly binds to SPRR2C, STAT1, and S100A7

**Fig. 4:**
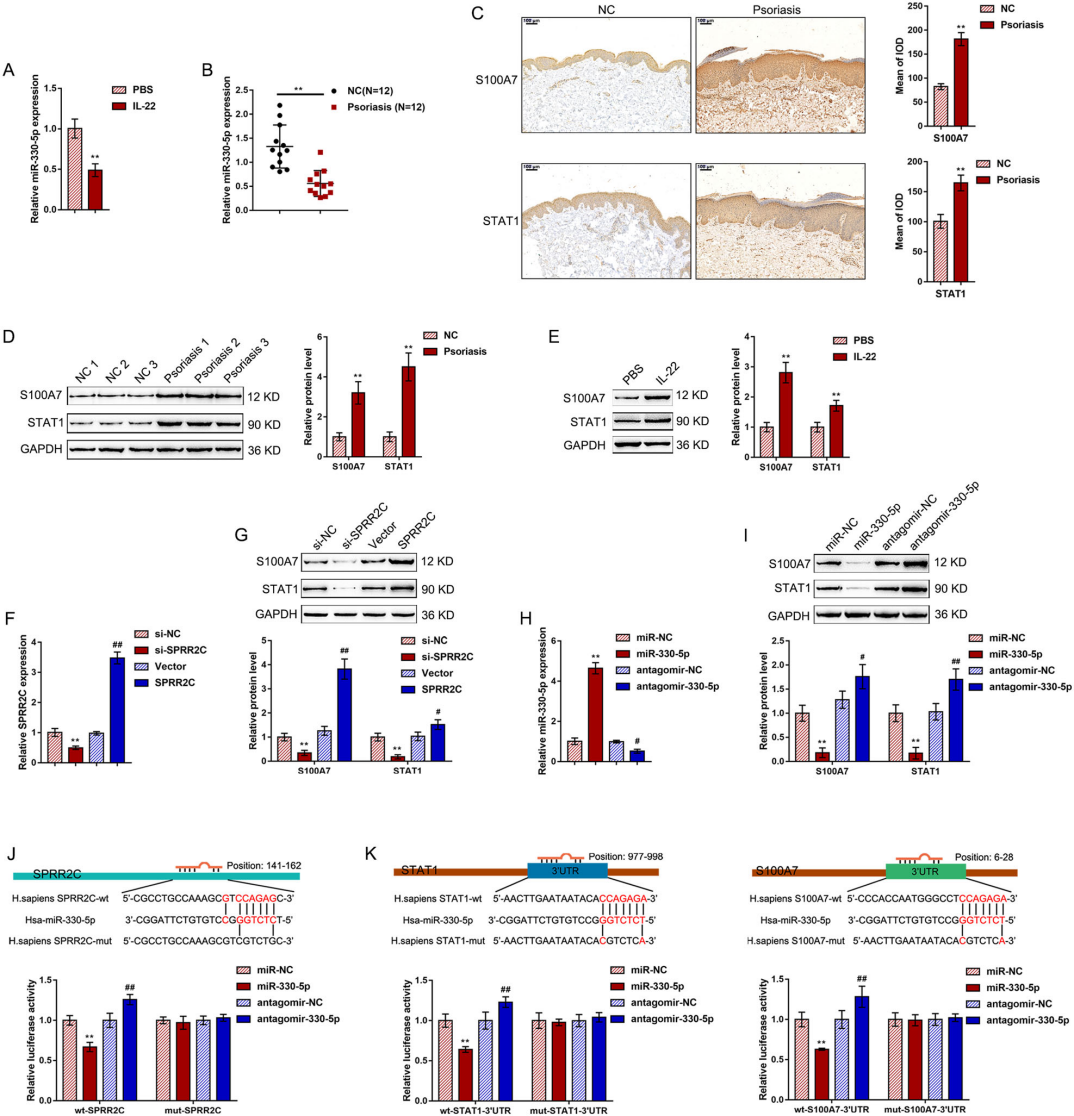
MiR-330 directly binds to SPRR2C, STAT1, and S100A7. **A** HaCaT cells were starved in serum-free DMEM for 24 h, treated with IL-22 (100 ng/ml) in serum-free DMEM for another 24 h or not treated, and then examined for the expression of miR-330 by real-time PCR. **B** The expression of miR-330 was determined in 12 psoriatic lesions and 12 nonlesion tissues by real-time PCR. **C** The protein content and distribution of STAT1 and S100A7 in the psoriatic lesion and nonlesion tissue samples were determined by immunohistochemistry (IHC) staining. **D** The protein levels of STAT1 and S100A7 in the psoriatic lesion and nonlesion tissue samples were determined by immunoblotting. **E** HaCaT cells were treated with IL-22 and examined for the protein levels of STAT1 and S100A7 by immunoblotting. **F** SPRR2C knockdown or overexpression was achieved in HaCaT cells by the transfection of si-SPRR2C or SPRR2C-overexpressing vector; the transfection efficiency was confirmed by real-time PCR. **G** HaCaT cells were transfected with si-SPRR2C or SPRR2C-overexpressing vector and examined for the protein levels of STAT1 and S100A7 by immunoblotting. **H** MiR-330 overexpression or inhibition was achieved by the transfection of miR-330 or antagomir-330-5p; the transfection efficiency was confirmed by real-time PCR. **I** HaCaT cells were transfected with miR-330 or antagomir-330-5p and examined for the protein levels of STAT1 and S100A7 by immunoblotting. **J**, **K** Wild-type and mutant-type SPRR2C, STAT1 3′UTR, and S100A7 3′UTR luciferase reporter vectors were constructed as described in “Materials and Methods”. These vectors were cotransfected into 293T cells with miR-330 or antagomir-330-5p, and the luciferase activity was determined. **P < 0.01, ^#^*P* < 0.05, ^##^*P* < 0.01.

Before screening for the downstream targets of miR-330, we first examined the expression of miR-330 in the IL-22-stimulated HaCaT cell lines and tissue samples. The expression of miR-330 was significantly downregulated in response to IL-22 treatment (Fig. 4A). Consistently, the expression of miR-330 was significantly reduced in the psoriatic lesions compared with the normal control skin tissue samples (Fig. 4B). Considering the role of SPRRC2 in proliferation, dedifferentiation, and innate immunity-related inflammation, we also examined the expression of SPRRC2 and miR-330 in IL-22-stimulated primary human keratinocytes. As shown in Supplementary Fig. S4A, B, in primary human keratinocytes, IL-22 stimulation upregulated SPRRC2 expression but downregulated miR-330-5p expression. In addition, since IL-17A plays a central role in psoriatic pathogenesis^38^, we examined the expression of SPRRC2 and miR-330 in HaCaT cells in response to IL-17A stimulation. As shown in Supplementary Fig. S4C, D, IL-17A stimulation of HaCaT cells significantly induced SPRRC2 expression but inhibited miR-330-5p expression.

Next, we screened GSE114286 for the differentially expressed genes negatively correlated with miR-330 (logFC > 2, *P* < 0.0005, *r* < −0.65) and positively correlated with SPRR2C (logFC > 2, *P* < 0.0005, *r* > 0.65); a total of eight candidate genes were obtained: S100A7, PARP14, OAS2, LAMP3, GBP5, STAT1, SPRR2A, and EPSTI1. After cross-check analysis of six datasets (GSE13355, GSE14905, GSE30999, GSE34248, GSE41662 GSE50790), STAT1 and S100A7 were significantly positively correlated with SPRR2C in all six datasets (Supplementary Table S4).

To further validate the correlation of STAT1 and S100A7 with SPRR2C in psoriasis, we next evaluated the STAT1 and S100A7 protein levels and distribution in the psoriatic lesional and normal control tissue samples. Figure 4C shows that there were more STAT1- and S100A7-positive areas in the psoriatic lesional tissue samples than in the normal control tissue samples. Moreover, the STAT1 and S100A7 protein contents were both significantly increased in the psoriatic lesions compared with the normal control tissues (Fig. 4D). In HaCaT cells, IL-22 treatment dramatically enhanced the STAT1 and S100A7 protein contents (Fig. 4E). These data indicate that, similar to SPRR2C, STAT1 and S100A7 are abnormally upregulated in psoriasis.

Next, we transfected si-SPRR2C or SPRR2C-overexpressing vector to achieve SPRR2C knockdown or overexpression in HaCaT cell lines and performed real-time PCR to verify the transfection efficiency (Fig. 4F). In HaCaT cells, the STAT1 and S100A7 protein contents were significantly enhanced by SPRR2C overexpression but inhibited by SPRR2C knockdown (Fig. 4G). We transfected miR-330 or antagomir-330-5p to achieve miR-330 overexpression or miR-330 inhibition and performed real-time PCR to verify the transfection efficiency (Fig. 4H). In HaCaT cells, miR-330 overexpression was significantly downregulated, whereas miR-330 overexpression upregulated the STAT1 and S100A7 protein levels (Fig. 4I). These data suggest that SPRR2C positively modulates the STAT1 and S100A7 protein levels, while miR-330 negatively modulates the STAT1 and S100A7 protein levels.

To investigate the putative miR-330 binding to SPRR2C, STAT1, and S100A7, we performed a luciferase reporter assay. According to “Materials and Methods”, we constructed two different types of SPRR2C, STAT1 3′UTR, and S100A7 3′UTR luciferase reporter vectors, wild-type, and mutant-type. We cotransfected these vectors in 293T cells with miR-330 or antagomir-330-5p and determined the luciferase activity. Figure 4J, K shows that the luciferase activity of wild-type reporter vectors, including wt-SPRR2C, wt-STAT1 3’UTR, and wt-S100A7 3′UTR, was dramatically downregulated by the overexpression of miR-330 but upregulated by the inhibition of miR-330; mutating the putative miR-330-binding site eliminated the alterations in the luciferase activity (Fig. 4J, K). In summary, miR-330 directly binds to SPRR2C, the STAT1 3′UTR, and the S100A7 3′UTR.

### Effects of miR-330 on STAT1 and S100A7 and IL-22-induced HaCaT cells

**Fig. 5:**
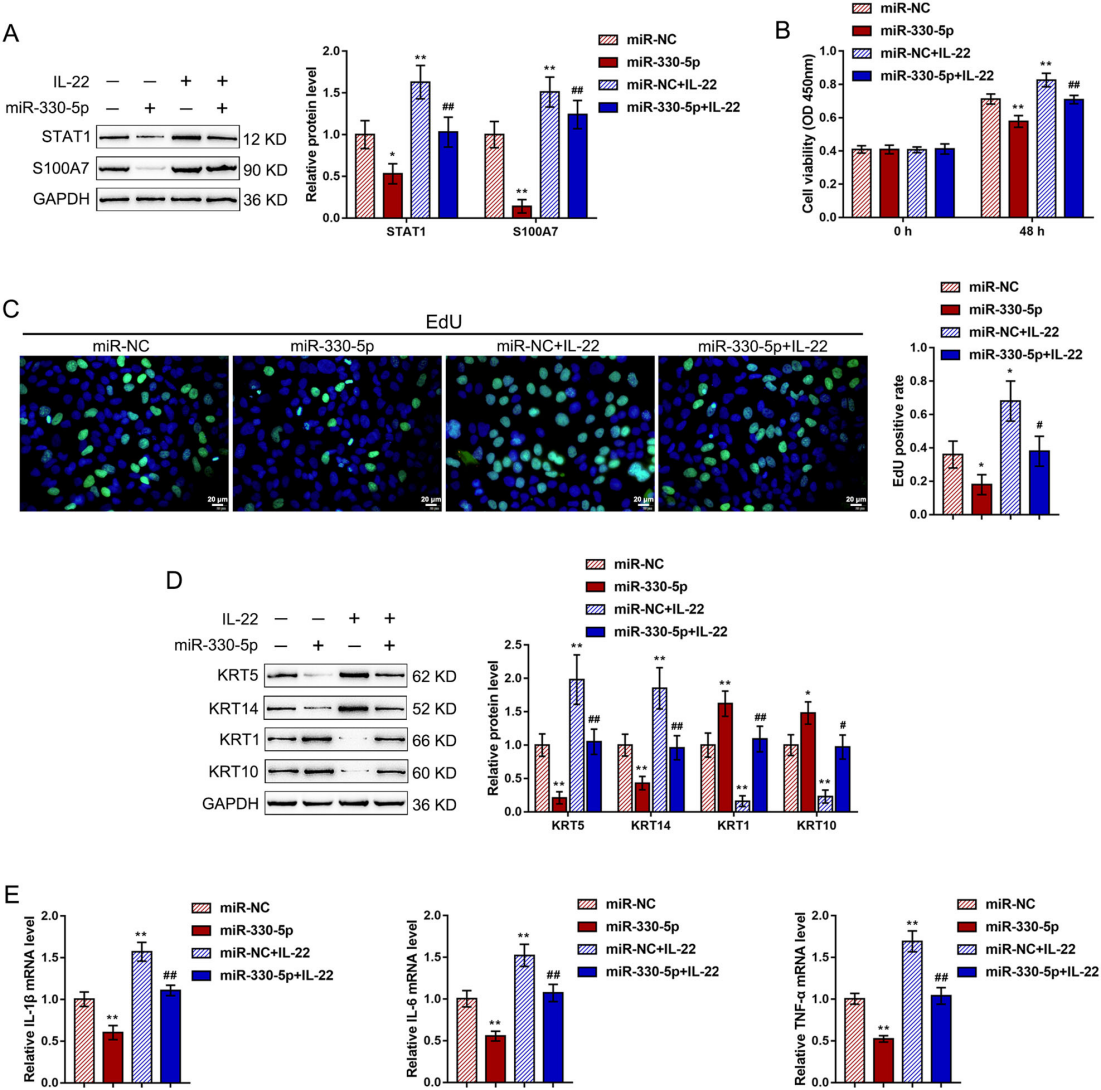
Effects of miR-330 on STAT1 and S100A7 and IL-22-induced HaCaT cells. HaCaT cells were transfected with miR-330-5p in the presence or absence or IL-22 and examined for (**A**) the protein levels of STAT1 and S100A7 by immunoblotting; **B** the cell viability by MTT assays; **C** DNA synthesis by EdU assays; **D** the protein levels of KRT5/14/1/10 by immunoblotting; **E** and the mRNA expression levels of IL-1β, IL-6, and TNF-α by real-time PCR. *P < 0.05, **P < 0.01, compared to the control group; ^#^*P* < 0.05, ^##^*P* < 0.01, compared to the miR-NC+IL-22 group.

After confirming miR-330 binding to SPRR2C, the STAT1 3′UTR, and the S100A7 3′UTR, we further investigated the in vitro roles of miR-330 in the IL-22-treated HaCaT cell lines. Consistent with earlier results, IL-22 treatment dramatically enhanced the STAT1 and S100A7 protein contents (Fig. 5A), promoted the cell viability (Fig. 5B) and DNA synthesis (Fig. 5C) of HaCaT cells, increased K5/14 while decreasing the K1/10 protein levels (Fig. 5D), and upregulated the mRNA expression levels of the inflammatory factors IL-1β, IL-6, and TNF-α (Fig. 5E); conversely, miR-330 overexpression exerted the opposite effects (Fig. 5A–E). Above all, miR-330 overexpression dramatically attenuated the effects of IL-22 stimulation (Fig. 5A–E).

### Dynamic effects of lncRNA SPRR2C and miR-330 on the STAT1- and S100A7- and IL-22-induced HaCaT cells

**Fig. 6:**
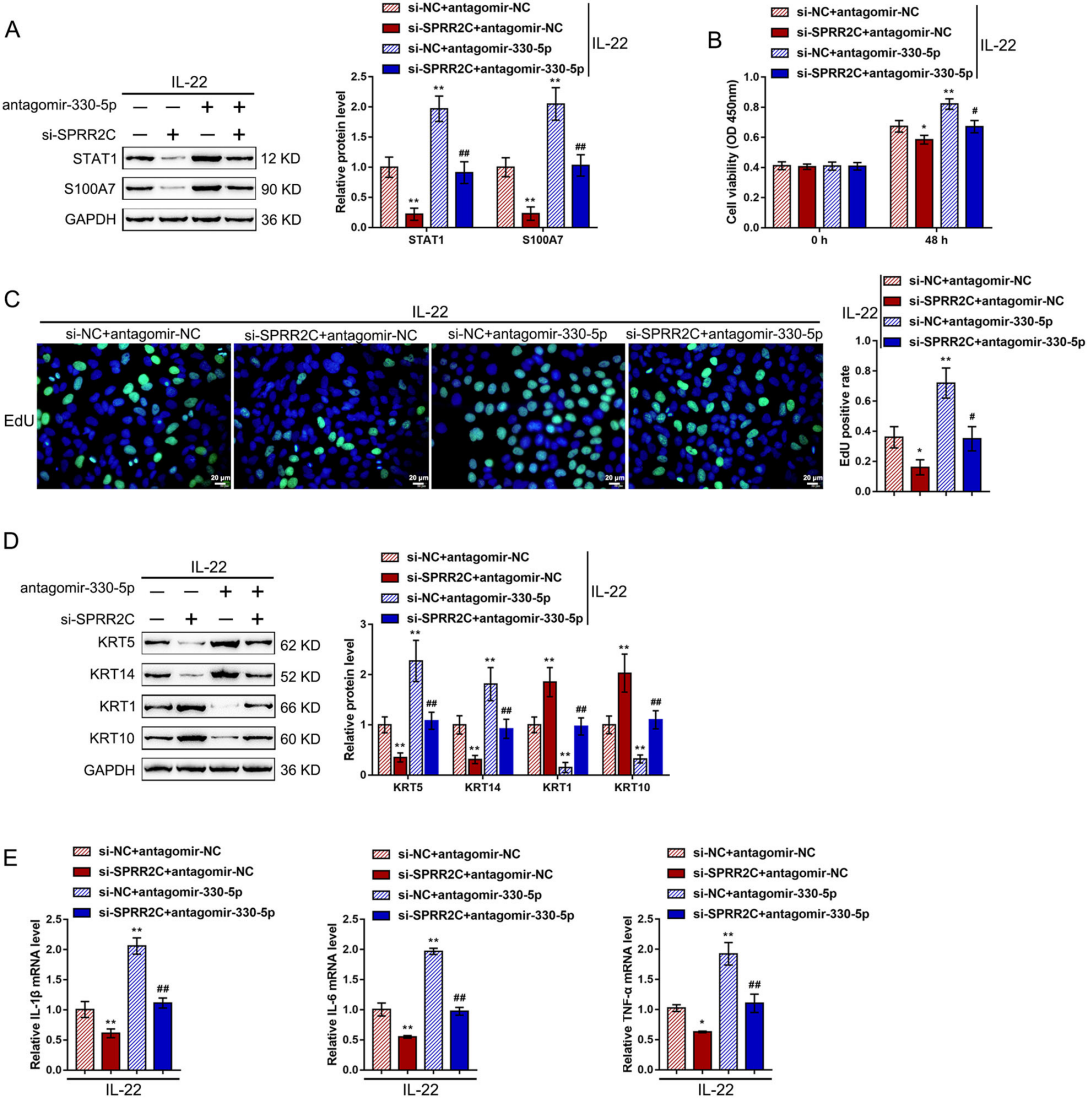
Dynamic effects of lncRNA SPRR2C and miR-330 on STAT1 and S100A7 and IL-22-induced HaCaT cells. HaCaT cells were cotransfected with si-SPRR2C and antagomir-330-5p under L-22 stimulation and examined for (**A**) the protein levels of STAT1 and S100A7 by immunoblotting; **B** the cell viability by MTT assays; **C** DNA synthesis by EdU assays; **D** the protein levels of KRT5/14/1/10 by immunoblotting; **E** and the mRNA expression levels of IL-1β, IL-6, and TNF-α by real-time PCR. *P < 0.05, **P < 0.01, compared to the control group; ^#^*P* < 0.05, ^##^*P* < 0.01, compared to the si-NC+ antagomir-330-5p group.

Next, the study investigated the dynamic effects of SPRR2C and miR-330 to evaluate whether SPRR2C could counteract the miR-330-mediated repression of STAT1 and S100A7, therefore modulating the IL-22-stimulated changes in HaCaT cell lines. We cotransfected HaCaT cell lines with si-SPRR2C and antagomir-330-5p under IL-22 stimulation and examined related indexes. Upon IL-22 stimulation, SPRR2C silencing strongly decreased the STAT1 and S100A7 protein contents (Fig. 6A), inhibited cell viability (Fig. 6B) and DNA synthesis (Fig. 6C), reduced the KRT5/14 protein contents, and increased the KRT1/10 protein contents (Fig. 6D), and downregulated IL-1β, IL-6, and TNF-α mRNA expression (Fig. 6E). MiR-330 inhibition exerted the opposite effects on these indexes (Fig. 6A–E). Above all, miR-330 inhibition could dramatically attenuate the effects of SPRR2C knockdown, suggesting that SPRR2C counteracts miR-330-induced repression upon STAT1 and S100A7 via competitive binding to miR-330, further modulating the IL-22-induced changes in HaCaT cells through miR-330/STAT1/S100A7. Moreover, in human keratinocytes, IL-22 induces neutrophilic chemoattractant molecules, such as CX3CL1, CXCL8, CXCL1, and CXCL16^39,40^. Thus, we examined the changes in the CX3CL1, CXCL8, CXCL1, and CXCL16 levels in the HaCaT cells transfected with si-NC or si-SPRRC2 in the presence or absence of IL-22 stimulation. As shown in Supplementary Fig. S5A–D, IL-22 stimulation significantly increased the CX3CL1, CXCL8, CXCL1, and CXCL16 levels; SPRRC2 silencing significantly reduced the levels of CX3CL1, CXCL1, and CXCL16 but not CXCL8.

### Expression and correlation of related factors in the tissue samples

**Fig. 7:**
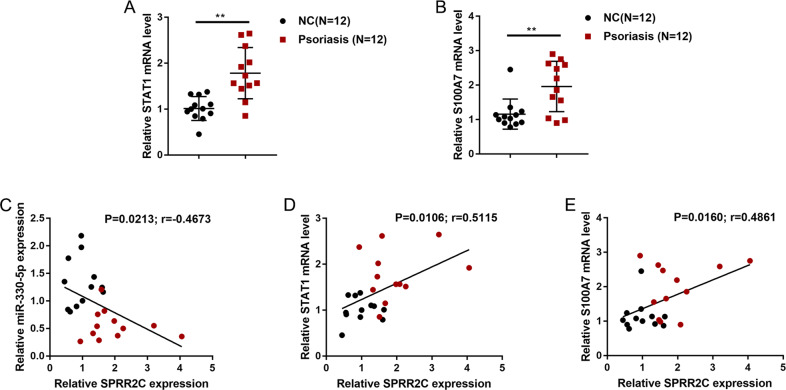
Expression and correlation of related factors in tissue samples. **A**, **B** The expression of STAT1 and S100A7 was determined in 12 psoriatic lesions and 12 nonlesion tissue samples by real-time PCR. **C**–**E** The correlation of SPRR2C, miR-330, STAT1, and S100A7 expression was analyzed by Pearson’s correlation analysis.

To further confirm the axis in vivo, we performed real-time PCR to examine the expression of STAT1 and S100A7 in 12 psoriatic lesions and 12 normal skin tissues. Figure 7A, B shows that STAT1 and S100A7 expression was significantly upregulated in the psoriatic lesional tissue samples compared with the normal skin tissue samples. In tissues, the expression of miR-330 had a negative correlation with the expression of SPRR2C (Fig. 7C), and the expression of SPRR2C had a positive correlation with the expression of STAT1 (Fig. 7D) and the expression of S100A7 (Fig. 7E).

## Discussion

Here, lncRNA SPRR2C was reported to be a hub gene exerting a critical effect on the pathogenesis of psoriasis and response to treatment using both WGCNA and differential expression analyses. The expression of SPRR2C was dramatically increased in the psoriatic lesion samples and in the HaCaT cell lines in response to IL-22 stimulation. After SPRR2C knockdown, IL-22-induced suppression of HaCaT proliferation, changes in the KRT5/14/1/10 protein levels, and suppression of IL-1β, IL-6, and TNF-α mRNA expression were substantially reversed. In the coexpression network with SPRR2C based on GSE114286, miR-330 was significantly negatively correlated with SPRR2C, while STAT1 and S100A7 were positively correlated with SPRR2C. By binding to miR-330, SPRR2C competed with STAT1 and S100A7 to counteract the miR-330-mediated suppression of STAT1 and S100A7. MiR-330 overexpression also reversed the IL-22-induced changes in HaCaT cell lines; in response to IL-22 treatment, miR-330 inhibition could significantly attenuate the effects of SPRR2C knockdown. In the tissue samples, STAT1 and S100A7 expression were significantly upregulated in the psoriatic lesion tissues. The expression of miR-330 exhibited a negative correlation with the expression of SPRR2C, while the expression of SPRR2C exhibited a positive correlation with the expression of STAT1 and S100A7.

Differential expression analysis can identify individual genes that are differentially expressed between cases and controls, while WGCNA can uncover additional biological pathways related to the pathogenesis of psoriasis and response to therapy. This study screened for lncRNAs both differentially expressed and in coexpression networks associated with psoriasis and response to treatment and identified lncRNA SPRR2C as a potential hub gene. We also inferred the function of genes in the coexpression module most significantly correlated to the pathological features of psoriasis (turquoise module) by testing these coexpression networks for GO enrichment analysis and found that they were significantly enriched in psoriatic pathogenesis-related biological processes, including the production of inflammatory factors (inflammation), endopeptidase activity (bactericidal), and bacterial lipopolysaccharide response (immunity). We identified a total of 81 lncRNAs in the turquoise module, which is consistent with another WGCNA on psoriasis, in which the majority of the genes in most of the modules dramatically associated with psoriatic lesions were lncRNAs^16^. This finding suggests that lncRNAs likely regulate coding genes in critical pathways, thus exerting a key effect on the pathogenesis of psoriasis.

In the intact human epidermis, the SPRR1 and 2 proteins are crosslinked to loricrin, keratins 1, 2, and 10 (KRT1/2/10), filaggrin, and elafin^41^. We found that the mRNA expression of SPRR1A and 2C was upregulated in atopic dermatitis-related skin lesions^42^. Previous studies reported significant upregulation of SPRR1 and 2 gene expression in inflammatory skin diseases^40^, which was explained as part of the response to diseases requiring rapid epidermal regeneration. SPRR gene expression is thought to play a critical role in cornified envelope biomechanical performance^43,44^. As previously reported, the SPRR2C protein was much more strongly increased in psoriatic lesions than in atopic dermatitis^45^ and is considered to be one of the epithelial host defense proteins^46^. In this study, lncRNA SPRR2C was regarded as a significantly upregulated lncRNA in psoriatic lesion tissues by both bioinformatics and experimental analyses. IL-22 stimulation of human keratinocyte HaCaT cells induced a series of changes, including enhanced cell proliferation, increased KRT5/14 protein levels, decreased KRT1/10 protein levels, and increased mRNA expression of the inflammatory factors IL-1β, IL-6, and TNF-α; in contrast, lncRNA SPRR2C knockdown significantly reversed these in vitro changes. IL-22 has been reported to stimulate IL-1β secretion through keratinocytes to promote inflammation involving the skin, therefore resulting in phenotypic alterations of keratinocytes^47^. Similarly, in this study, knocking down lncRNA SPRR2C reversed IL-22-induced inflammation involving the skin and phenotypic changes in keratinocytes.

It has been demonstrated that lncRNAs function as a critical class of ceRNAs (competing endogenous RNAs)^48^. LncRNAs can also act as molecular sponges for a miRNA through their miRNA-binding sites, thereby derepressing all target genes of the miRNA family. In the coexpression network of lncRNA SPRR2C based on GSE114286, five miRNAs were found to be negatively correlated with lncRNA SPRR2C (miR-330, miR-663a, miR-4489, miR-4720, and miR-4725). Among these five miRNAs, miR-330 has been reported by our previous study to target CTNNB1, thus inhibiting the inducible effect of IL-22 on the proliferation of keratinocytes^37^. Additionally, in this coexpression network of lncRNA SPRR2C based on GSE114286, two coding genes, STAT1 and S100A7, were found to have a positive correlation with lncRNA SPRR2C while having a negative correlation with miR-330. STAT1 activation is critical for IFN-γ-induced upregulation of the expression of keratin 17 (K17), a cytoskeletal protein overexpressed in the psoriatic lesional epidermis but not found in the healthy epidermis and considered a hallmark of psoriasis^49,50^. S100A7 (S100 calcium-binding protein A7), also called psoriasin, is a protein encoded by the S100A7 gene in the human body and is markedly overexpressed in the skin lesions of patients with psoriasis^51^. Consistent with these online data and previous studies, the protein levels of both STAT1 and S100A7 were significantly increased in the psoriatic lesion tissues and increased by IL-22 stimulation in HaCaT cells. More importantly, SPRR2C positively regulated STAT1 and S100A7 expression, while miR-330 negatively regulated STAT1 and S100A7 expression. Through binding to miR-330, SPRR2C competed with STAT1 and S100A7, therefore counteracting the miR-330-mediated suppression of STAT1 and S100A7.

Previously, our study reported that miR-330 targets CTNNB1 to inhibit the inducible effects of IL-22 on the proliferation of keratinocytes^37^. Other studies also indicated the effect of miR-330 on the proliferation, differentiation, and migration of keratinocytes. Kim et al.^52^ demonstrated that miR-330-5p suppresses the capacity of keratinocytes to proliferate and migrate by binding to Pdia3 and inducing G0/G1 cell cycle arrest. Similar to Pdia3 knockdown, SRPR knockdown also inhibited the proliferation of keratinocytes, while miR-330-5p binds to SRPR in its 3′UTR to directly downregulate its expression^53^. Herein, miR-330 overexpression not only inhibited the inducible effects of IL-22 on the proliferation of keratinocytes but also reversed the effects of IL-22 on KRT5/14/1/10 and the inflammatory factors IL-1β, IL-6, and TNF-α. In summary, miR-330 overexpression suppressed IL-22-stimulated inflammatory changes in vitro. Since lncRNA SPRR2C targets miR-330 to inhibit its expression, we further investigated the dynamic effects of lncRNA SPRR2C/miR-330 on downstream STAT1 and S100A7 and the IL-22 effects on HaCaT cells. In contrast to SPRR2C knockdown, miR-330 inhibition aggravated the IL-22-induced inflammatory changes in HaCaT cells. Above all, miR-330 inhibition markedly attenuated the effects of lncRNA SPRR2C knockdown, suggesting that lncRNA SPRR2C affects IL-22-induced HaCaT cell lines by targeting miR-330.

In human keratinocytes, IL-22 induces neutrophilic chemoattractant molecules, such as CX3CL1, CXCL8, CXCL1, and CXCL16^39,40^. In this study, we also observed consistent results; in IL-22-stimulated HaCaT cells, the levels of CX3CL1, CXCL8, CXCL1, and CXCL16 were all significantly induced compared with those of the nonstimulation group. However, after silencing of SPRR2C, only the levels of CX3CL1, CXCL1, and CXCL16, reduce but not that of CXCL8, were significantly reduced. Previously, Sestito et al.^54^ demonstrated that S100A7 induction by IL-22 is independent of STAT3, whereas CXCL8 and CXCL1 induction is STAT3 dependent. Conversely, Liu et al.^55^ reported that topoisomerase inhibitor-promoted CXCL1 was mediated by the JAK/STAT1 pathway. Moreover, STAT1 controls the recruitment of Th1 cells through the induction of CXCs, including CXCL16^56^. Similarly, inhibitors of JAK/STAT, NF-κB, and AP-1 significantly reduced CX3CL1 and CX3CR1 expression^57^. As we have revealed, the miR-330/STAT1/S100A7 axis is downstream of SPRR2C. Thus, the changes in CX3CL1, CXCL1, and CXCL16 levels following SPRR2C silencing might also be mediated by STAT1. The activation of STAT1 and its downstream signaling pathways contribute to the recruitment of Th1 cells, sensitizing keratinocytes to inflammatory factors and aggravating the inflammatory response^58–60^ by increasing Th1-type cytokines, including IL-1β and TNF-α. Subsequently, elevated TNF-α increases the production of Th2-type IL-6^61,62^. Thus, the effects of miR-330 on these cytokines might also be attributed to STAT1 and its downstream signaling pathways.

Finally, in tissue samples, the expression of STAT1 and S100A7 was significantly upregulated in the psoriatic lesion tissues. The expression of miR-330 had a negative correlation with the expression of lncRNA SPRR2C, while the expression of lncRNA SPRR2C had a positive correlation with the expression of STAT1 and S100A7, further supporting the existence of the lncRNA SPRR2C/miR-330/STAT1/S100A7 axis in vivo.

In conclusion, SPRR2C modulates the IL-22-stimulated HaCaT cell phenotype through the miR-330/STAT1/S100A7 axis. WGCNA might uncover additional biological pathways that are crucial in the pathogenesis and response to the treatment of psoriasis.

## Supplementary information

Suplementary figure1

Suplementary figure2

Suplementary figure3

Suplementary figure4

Suplementary figure5

Suplementary figure6

Suplementary table S1

Suplementary table S2

Suplementary table S3

Suplementary table S4
