# Supplementary material for: Weighted gene coexpression network and experimental analyses identify lncRNA SPRR2C as a regulator of the IL-22-stimulated HaCaT cell phenotype through the miR-330/STAT1/S100A7 axis
Source: Cell Death Dis. 2021 Jan 15;12(1):86. doi: 10.1038/s41419-020-03305-z (PMC7810847; doi:10.1038/s41419-020-03305-z)
Supplement: Supplementary file 1 — Suplementary figure1 [file 41419_2020_3305_MOESM1_ESM.docx]

**Fig. S1** **Weighted gene coexpression network analysis (WGCNA) based on online data for gene modules related to the histopathological features of psoriasis** (A) Variance analysis was performed on the 22188 genes differentially expressed in 170 psoriatic skin lesions and normal control tissues reported by GSE30999. Sample dendrograms and trait heatmaps are shown. Light blue modules represent lesion skin samples, and gray modules represent nonlesion skin samples. (B) The top 5000 differentially expressed genes were applied for WGCNA. The cluster showed 11 gene coexpression modules formed by these 5000 differentially expressed genes. Different gene coexpression modules are represented in red, green-yellow, blue, yellow, pink, green, azure, red-brown, purple, black, and magenta. Gray represents genes not successfully clustered. (C) Analysis of network topology for various soft-thresholding powers. The left panel shows the scale-free fit index (y-axis), namely, signed R^2^, as a function of the soft-thresholding power (x-axis). The sign of the scale-free model fitting index R^2^ is determined by subtracting the sign of the slope, cex1=0.9. The right panel displays the mean connectivity (degree, y-axis) as a function of the soft-thresholding power (x-axis). When the weight is taken to 6 (β=6), the evaluation parameter of the scale-free network can be close to 0.9. Weight 6, thus, was used in subsequent analysis. (D) β=6 was selected to calculate the network topology overlap topological overlap matrix (TOM). The hierarchical clustering method was used to obtain the network heatmap plot. Each row and column in the heatmap corresponds to one gene. The darker the color, the higher the correlation or topological overlap between genes is and the closer the relationship between genes is. The left and top of the figure are the gene coexpression modules shown in the cluster dendrogram. (E) Heatmap showing the correlation between the gene coexpression modules. (F) The correlations between gene coexpression modules and pathological characteristics (psoriatic lesion, LS, and nonlesion, NL) were analyzed and are shown in a module-trait relationship heatmap. The turquoise module has a very significant positive correlation with the clinical features of psoriasis (r = 0.92, p = 3e-88) and a strong negative correlation with normal control tissues. Therefore, we further analyzed the turquoise module and found that the gene module contains 81 lncRNAs.

**Fig. S2 Differentially expressed abundant lncRNAs in psoriasis were screened using multiple sets of Gene Expression Omnibus (GEO) chips and RNA-seq data.** (A) Schematic diagram showing the selection process using 2 RNA-seq datasets and 6 microarray expression profiles from GEO. After cross-check analysis, the following 4 lncRNAs were regarded as differentially expressed abundant lncRNAs: SPRR2C, TMEM254-AS1, EPB41L4A-AS1, and SH3PXD2A-AS1. The expression of SPRR2C, TMEM254-AS1, EPB41L4A-AS1, and SH3PXD2A-AS1 in 92 psoriatic lesions and 82 normal biopsy samples according to the E-GEOD-54456 dataset (B), in 9 normal skin samples and 18 psoriatic lesion skin samples according to the GSE114286 (C), in 58 psoriatic lesions and 64 normal skin samples according to GSE13355 (D), in 30 psoriatic lesions and 30 normal skin samples according to GSE14905 (E), in skin lesions and peripheral normal skin samples from 85 patients with moderate and severe psoriasis according to GSE30999 (F), in lesion and nonlesion control skin samples obtained from 14 patients with psoriasis by perforated biopsy according to GSE34248 (G), and in lesion and nonlesion control skin samples obtained from 24 patients with psoriasis by perforated biopsy according to GSE41662 (H). (I) A total of nine data sets—8 GEO datasets (GSE114286, GSE13355, GSE14905, GSE30999, GSE34248, GSE41662, GSE50790, GSE6710) and an EBI (E-GEOD-54456)—were used to draw a clustering heatmap showing the differentially expressed genes between the psoriatic lesion skin tissues and nonlesion control skin tissues (logFC of the most significantly differentially expressed genes).

**Fig. S3 SPRR2C is a psoriatic lesion-specific lncRNA related to drug sensitivity in treatment** (A) After cross-check analysis, SPRR2C was among the 81 lncRNAs screened by WGCNA and among the 4 lncRNAs selected by online dataset analysis. Thus, SPRR2C was selected for further experiments. (B) SPRR2C expression in nonlesions, lesions without treatment, and lesions on days 1, 3, and 1/2/4/12 after 10 mg of topatinib treatment according to GSE69967. (C) SPRR2 expression in nonlesions, lesions without treatment, placebo-treated lesions, and Il-17 receptor antagonist brodalumab (140, 350, or 700 mg)-treated lesions according to GSE53552. (D) Fifteen patients with moderate to severe psoriasis received 50 mg etanercept treatment. SPRR2 expression in nonlesions, lesions without treatment, and lesions in week 1/2/4/12 after treatment from responders (left panel) and nonresponders (right panel) according to GSE11903. (E) SPRR2 expression in nonlesions, lesions treated with the CD11a antibody drug efalizumab, lesions with relapse, and lesions without relapse according to GSE30768. (F) According to GSE85034 reporting the differentially expressed genes in lesion and nonlesion tissues from patients with moderate to severe psoriasis who received adalimumab and methotrexate treatment (at 1, 2, 4, and 16 weeks after treatment), the correlation between SPRR2C expression and PASI was analyzed using Pearson’s and Spearman’s correlation analyses.

**Fig. S4 Expression of SPRR2C and miR-330 in cells in response to different stimuli** (A-B) The expression of SPRR2C and miR-330 in primary keratinocytes treated with PBS or IL-22. (C-D) The expression of SPRR2C and miR-330 in HaCaT cells treated with PBS or IL-17A.

**Fig. S5 CXCL1, CXCL8, CXCL16, and CX3CL1 mRNA levels in HaCaT cells transfected with si-NC or si-SPRR2C in the presence or absence of IL-22.**
