# Supplementary figures and images for: Weighted gene coexpression network and experimental analyses identify lncRNA SPRR2C as a regulator of the IL-22-stimulated HaCaT cell phenotype through the miR-330/STAT1/S100A7 axis
Source: Cell Death Dis. 2021 Jan 15;12(1):86. doi: 10.1038/s41419-020-03305-z (PMC7810847; doi:10.1038/s41419-020-03305-z)

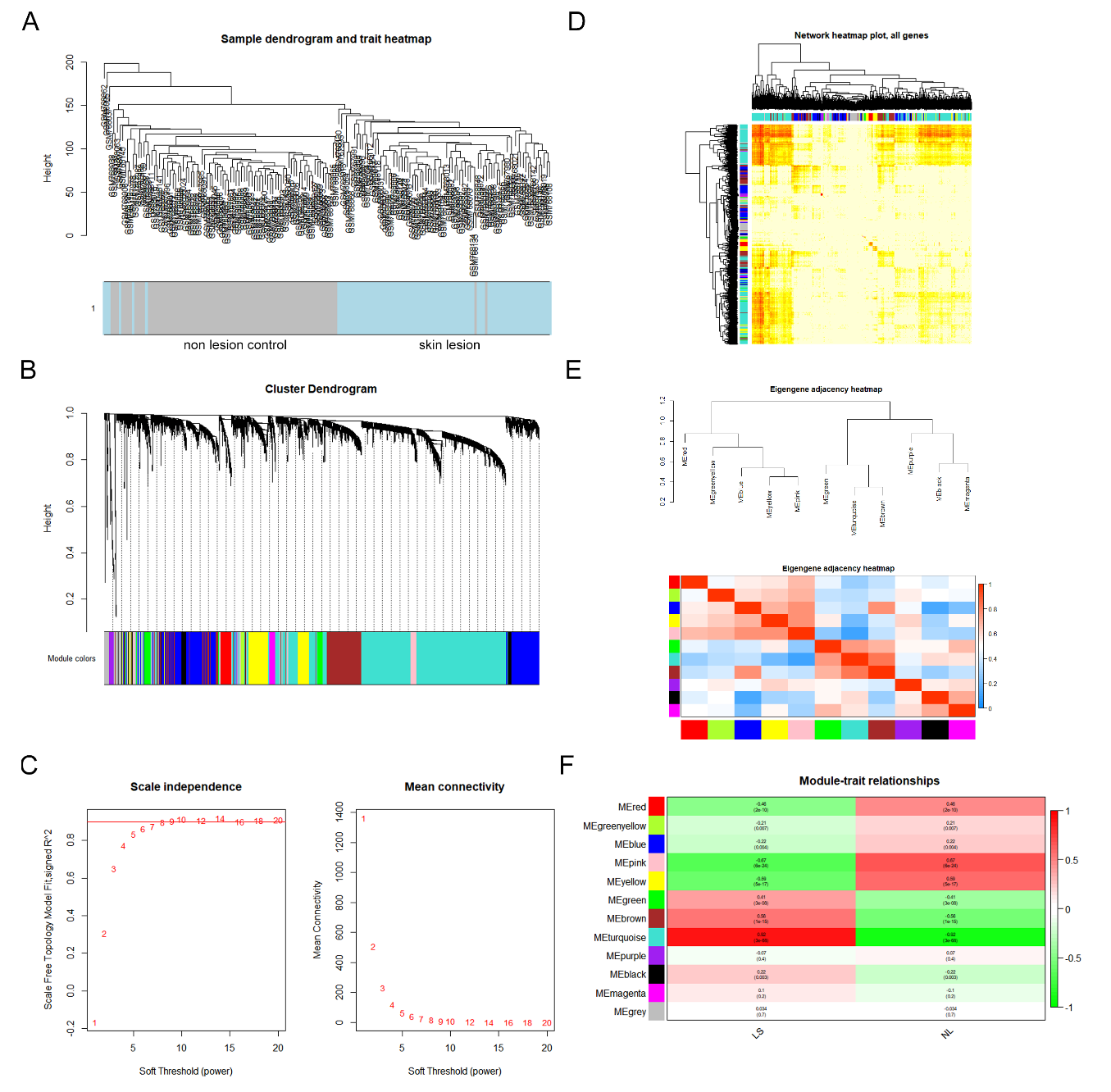

Supplement: Supplementary file 2 — Suplementary figure2 [file 41419_2020_3305_MOESM2_ESM.tif]

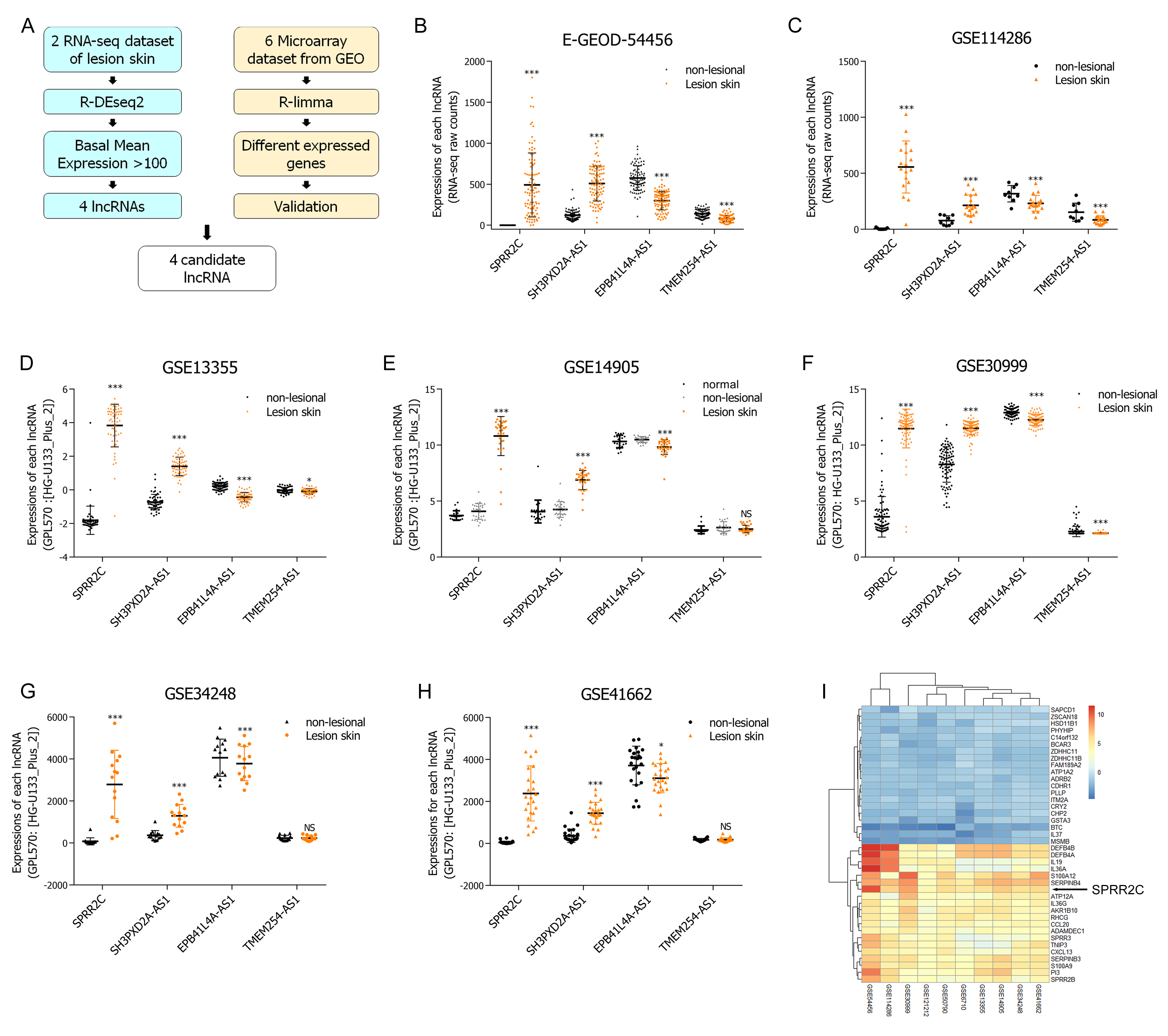

Supplement: Supplementary file 3 — Suplementary figure3 [file 41419_2020_3305_MOESM3_ESM.tif]

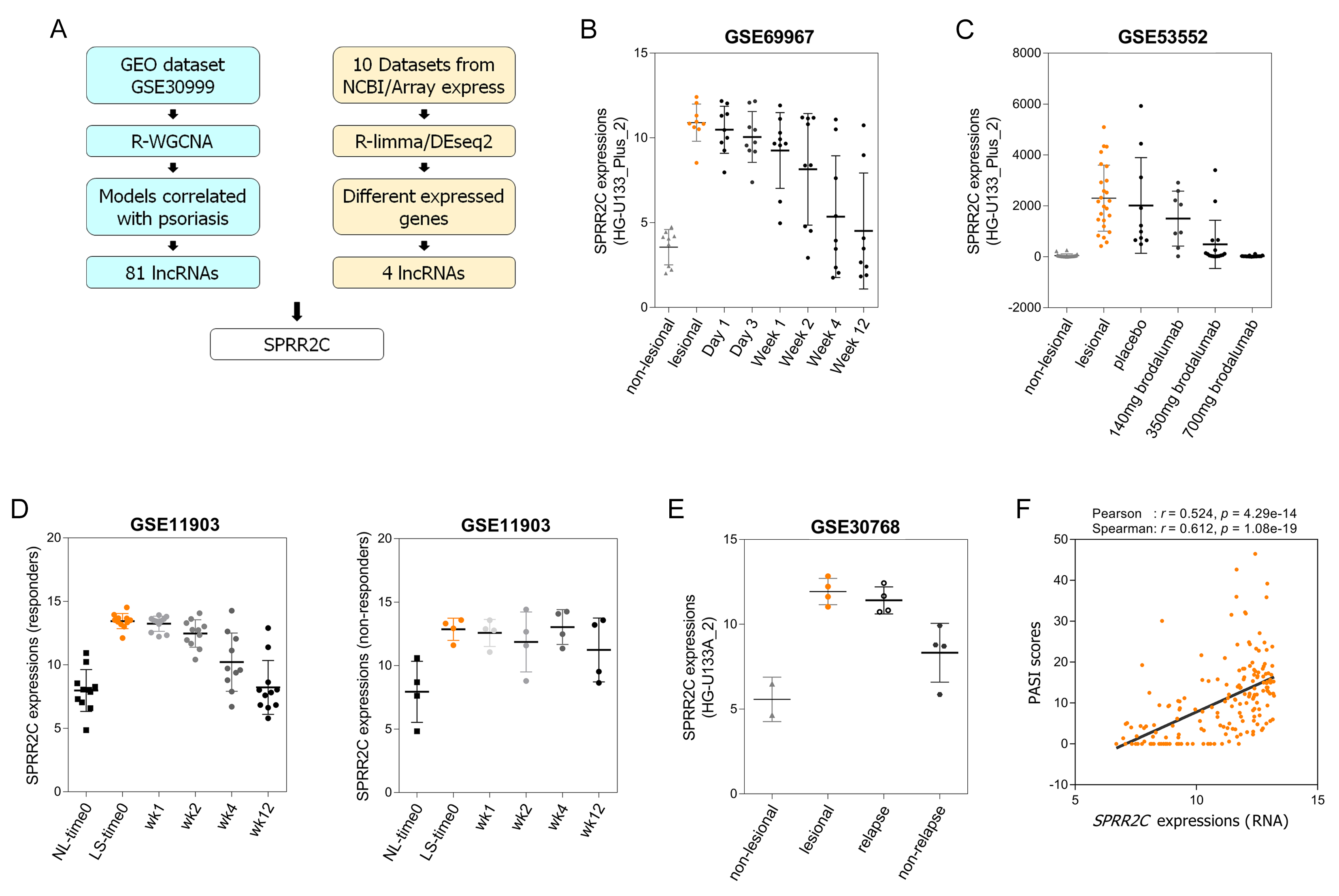

Supplement: Supplementary file 4 — Suplementary figure4 [file 41419_2020_3305_MOESM4_ESM.tif]

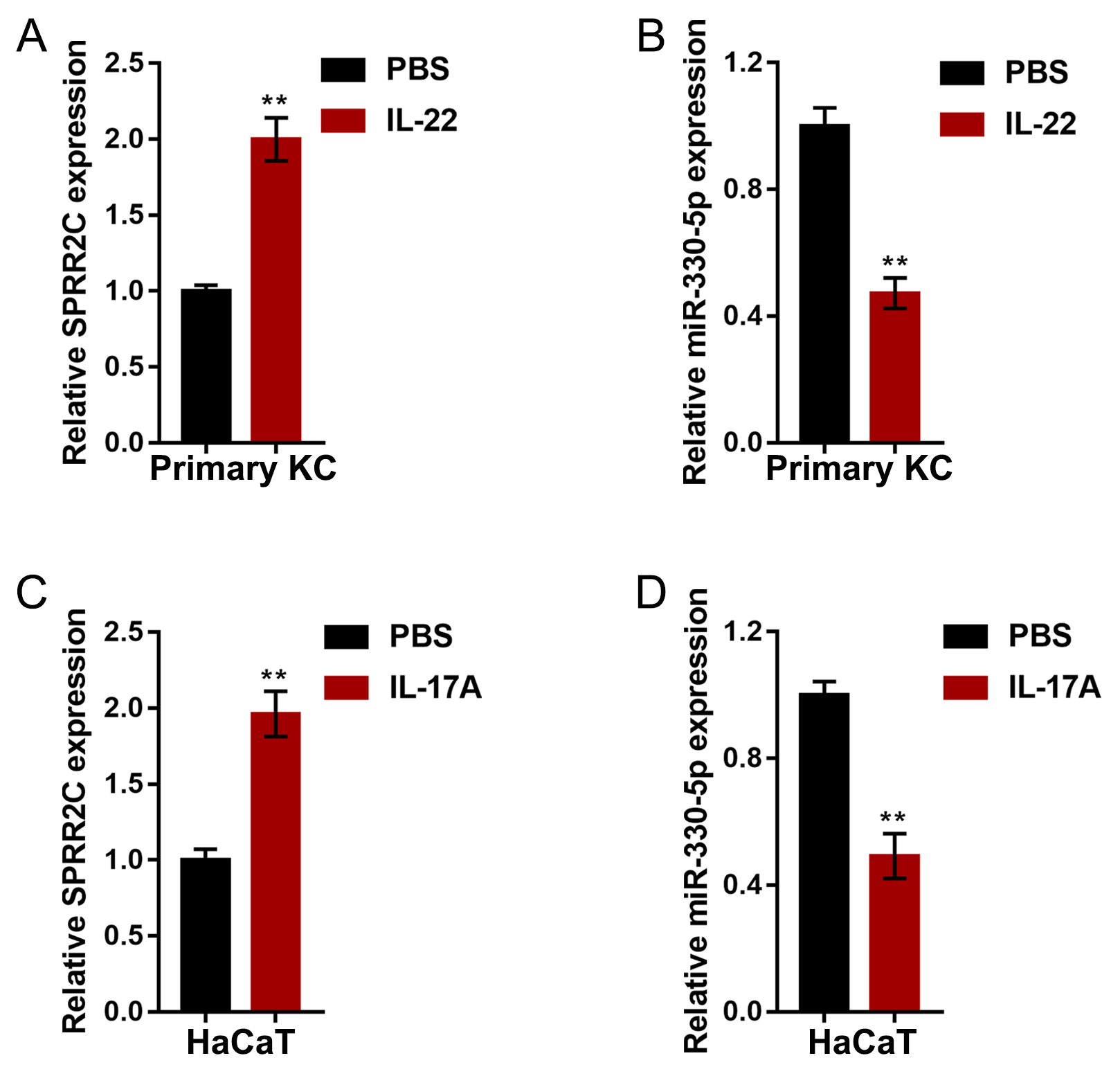

Supplement: Supplementary file 5 — Suplementary figure5 [file 41419_2020_3305_MOESM5_ESM.tif]

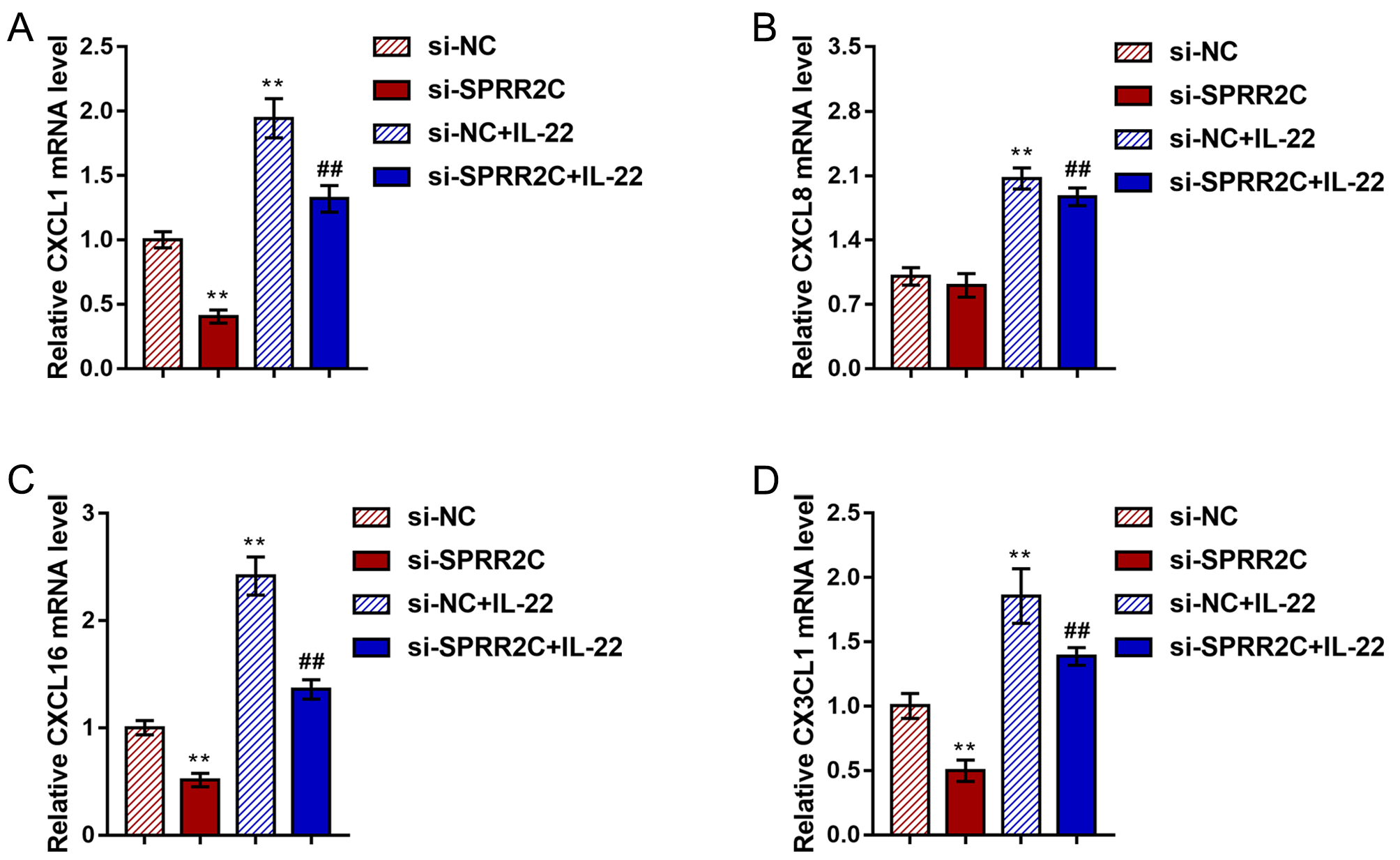

Supplement: Supplementary file 6 — Suplementary figure6 [file 41419_2020_3305_MOESM6_ESM.tif]
