# Supplementary material for: Weighted gene coexpression network and experimental analyses identify lncRNA SPRR2C as a regulator of the IL-22-stimulated HaCaT cell phenotype through the miR-330/STAT1/S100A7 axis
Source: Cell Death Dis. 2021 Jan 15;12(1):86. doi: 10.1038/s41419-020-03305-z (PMC7810847; doi:10.1038/s41419-020-03305-z)
Supplement: Supplementary file 7 — Suplementary table S1 [file 41419_2020_3305_MOESM7_ESM.docx]

**Table S1 the primer sequence**

| Name | Forward | Reverse |
| --- | --- | --- |
| RT-PCR  SPRR2C | CAGGACCACGAAAGGATAGGGA | GAAACATTTTGCTGTCGGGGA |
| RT-PCR  GAPDH | ACAGCCTCAAGATCATCAGC | GGTCATGAGTCCTTCCACGAT |
| RT-PCR  IL-1β | CTCCGACCACCACTACAGCAAG | TGGGCAGGGAACCAGCATC |
| RT-PCR  IL-6 | CAATGAGGAGACTTGCCTGGTG | GCTGGCATTTGTGGTTGGG |
| RT-PCR  TNF-a | CCCGAGTGACAAGCCTGTAGCC | CCCTTGAAGAGGACCTGGGAGTAGAT |
| RT-PCR  MiR-330-5p | RT:GTCGTATCCAGTGCGTGTCGTGGAGTCGGCAATTGCACTGGATACGACGCCTAA  F:GTCTCTGGGCCTGTGTC | R:CAGTGCGTGTCGTGGA |
| RT-PCR  STAT1 | CAGCTTGACTCAAAATTCCTGGA | TGAAGATTACGCTTGCTTTTCCT |
| RT-PCR  S100A7 | ACGTGATGACAAGATTGACAAGC | GCGAGGTAATTTGTGCCCTTT |
| Si-SPRR2C  mix | 1-F:UCAAAAUAAUUCUUUGAUGTT  2-F:GCCAGCAGAAAUAUCCUCUTT  3-F:CCUGUGAUCUGCCCAUGAUTT | 1-R:CAUCAAAGAAUUAUUUUGAUG  2-R:AGAGGAUAUUUCUGCUGGCTT  3-R:AUCAUGGGCAGAUCACAGGTT |
| Si-NC | UUCUCCGAACGUGUCACGUTT | ACGUGACACGUUCGGAGAATT |
| SPRR2C overexpression vector | ctaccggactcagatctcgagAGAGGTTCTCAGCCTCATGACTCC | gtaccgtcgactgcagaattcTAGGCATAGCTACTTTATTCAGGGAG |
| Wt-SPRR2C 3’UTR | aattctaggcgatcgctcgagAGAGGTTCTCAGCCTCATGACTCC | attttattgcggccagcggccgcTGCTCTTGGGTGGACACTTTG |
| mut-SPRR2C 3’UTR | AAAGCgtcgtctgCCATGTCCACCCCTGAAGTG | CATGGcagacgacGCTTTGGCAGGCGGCACACA |
| Wt-STAT1 3’UTR | aattctaggcgatcgctcgagACTCATTCAAAAGTTGAAATTAACCATA | attttattgcggccagcggccgcTACAGGAGAGAGAATGGAACCATTC |
| mut-STAT1 3’UTR | ACAcgtctcaTAATATGAGAATCAGATCATTTCAAAACTC | CATATTAtgagacgTGTATTATTCAAGTTGTCAGTTACTGCTT |
| Wt-S100A7 3’UTR | TCGAGGACCCAGCCCCACCAATGGGCCTCCAGAGACCCCAGGAGGTACCGC | GGCCGCGGTACCTCCTGGGGTCTCTGGAGGCCCATTGGTGGGGCTGGGTCC |
| mut-S100A7 3’UTR | TCGAGGACCCAGCCCCACCAATGGGCCTCGTCTCACCCCAGGAGGTACCGC | GGCCGCGGTACCTCCTGGGGTGAGACGAGGCCCATTGGTGGGGCTGGGTCC |
| Agomir-330-5p | UCUCUGGGCCUGUGUCUUAGGC | CUAAGACACAGGCCCAGAGAUU |
| Agomir-NC | UCACAACCUCCUAGAAAGAGUAGA | UCUACUCUUUCUAGGAGGUUGUGA |
| Antagomir-330-5p | GCCUAAGACACAGGCCCAGAGA |  |
| Antagomir-NC | UCUACUCUUUCUAGGAGGUUGUGA |  |
