# Supplementary material for: Weighted gene coexpression network and experimental analyses identify lncRNA SPRR2C as a regulator of the IL-22-stimulated HaCaT cell phenotype through the miR-330/STAT1/S100A7 axis
Source: Cell Death Dis. 2021 Jan 15;12(1):86. doi: 10.1038/s41419-020-03305-z (PMC7810847; doi:10.1038/s41419-020-03305-z)
Supplement: Supplementary file 8 — Suplementary table S2 [file 41419_2020_3305_MOESM8_ESM.docx]

Table S2 Information of dataset used in the study

|  | |  |  | |  | |  | |  | |
| --- | --- | --- | --- | --- | --- | --- | --- | --- | --- | --- |
| **Accessions** | **Tissue Type** | | | **Sample Size** | | **Array Type/Platform** | | **PubMed Reference ID** | |  |
| E-GEOD-54456 | PP | | | 92 | | RNA-seq/Illumina Genome Analyzer | | 24441097 | |  |
|  | PN | | | 83 | |  | |  | |  |
| GSE114286 | PP | | | 18 | | RNA-seq/Illumina Genome Analyzer | | 30341238 | |  |
|  | NN | | | 9 | |  | |  | |  |
| GSE13355 | PP | | | 58 | | Microarray/HG-U133_Plus_2 | | 19169254 | |  |
|  | PN | | | 58 | |  | |  | |  |
|  | NN | | | 64 | |  | |  | |  |
| GSE14905 | PP | | | 30 | | Microarray/HG-U133_Plus_2 | | 18648529 | |  |
|  | PN | | | 30 | |  | |  | |  |
| GSE30999* | PP | | | 85 | | Microarray/HG-U133_Plus_2 | | 22763790 | |  |
|  | PN | | | 85 | |  | |  | |  |
| GSE34248 | PP | | | 14 | | Microarray/HG-U133_Plus_2 | | 23308107 | |  |
|  | PN | | | 14 | |  | |  | |  |
| GSE41662 | PP | | | 24 | | Microarray/HG-U133_Plus_2 | | 23308107 | |  |
|  | PN | | | 24 | |  | |  | |  |
| GSE50790 | PP | | | 4 | | Microarray/HG-U133_Plus_2 | | 22479649 | |  |
|  | PN | | | 4 | |  | |  | |  |
| GSE6710 | PP | | | 13 | | Microarray/HG-U133A | | 16858420 | |  |
|  | PN | | | 13 | |  | |  | |  |
| GSE69967 | PP | | | 12 | | Microarray/HG-U133A | | 27059729 | |  |
|  | PN | | | 12 | |  | |  | |  |
|  | 12 weeks of Tofacitinib, PP | | | 8 | |  | |  | |  |
| GSE11903 | PP | | | 15 | | Microrarry/HG-U133A_2 | | 19895991 | |  |
|  | PN | | | 15 | |  | |  | |  |
|  | 12 weeks of efalizumab, PP | | | 15 | |  | |  | |  |
| GSE53552 | PP | | | 25 | | Microarray/HG-U133A | | 24646743 | |  |
|  | PN | | | 24 | |  | |  | |  |
|  | 43 days of 700 mg brodalumab, PP | | | 8 | |  | |  | |  |
| GSE30768 | PN | | | 2 | | Microrarry/HG-U133A_2 | | 22348003 | |  |
|  | PP | | | 4 | |  | |  | |  |
|  | 12 post-treatment, P | | | 4 | |  | |  | |  |
|  | Relapse, P | | | 4 | |  | |  | |  |

PP: Lesion skin

PN: Uninvolved Lesion skin

NN: Healthy Control

* Used for WGCNA analysis
