# Supplementary material for: Weighted gene coexpression network and experimental analyses identify lncRNA SPRR2C as a regulator of the IL-22-stimulated HaCaT cell phenotype through the miR-330/STAT1/S100A7 axis
Source: Cell Death Dis. 2021 Jan 15;12(1):86. doi: 10.1038/s41419-020-03305-z (PMC7810847; doi:10.1038/s41419-020-03305-z)
Supplement: Supplementary file 9 — Suplementary table S3 [file 41419_2020_3305_MOESM9_ESM.docx]

Table S3. miRNAs negatively correlated with SPRR2C according to GSE114286

| miRNA | r | p-value |
| --- | --- | --- |
| MIR330 | -0.60748 | 0.000778 |
| MIR4489 | -0.52295 | 0.00513 |
| MIR4720 | -0.58942 | 0.001216 |
| MIR4725 | -0.52398 | 0.005027 |
| MIR663A | -0.5127 | 0.006248 |
