# Supplementary material for: Weighted gene coexpression network and experimental analyses identify lncRNA SPRR2C as a regulator of the IL-22-stimulated HaCaT cell phenotype through the miR-330/STAT1/S100A7 axis
Source: Cell Death Dis. 2021 Jan 15;12(1):86. doi: 10.1038/s41419-020-03305-z (PMC7810847; doi:10.1038/s41419-020-03305-z)
Supplement: Supplementary file 10 — Suplementary table S4 [file 41419_2020_3305_MOESM10_ESM.docx]

Table S4. STAT1 and S100A7 positively correlated with SPRR2C in all the 6 datasets

|  | S100A7 | | STAT1 | |
| --- | --- | --- | --- | --- |
|  | r | p | r | p |
| GSE13355 | 0.823284 | 1.21E-45 | 0.870348 | 1.19E-56 |
| GSE14905 | 0.821413 | 3.33E-21 | 0.89225 | 2.36E-29 |
| GSE30999 | 0.798351 | 7.59E-39 | 0.839228 | 2.60E-46 |
| GSE34248 | 0.883391 | 4.80E-10 | 0.782381 | 8.72E-07 |
| GSE41622 | 0.850726 | 1.93E-14 | 0.793176 | 1.80E-11 |
| GSE50790 | 0.863069 | 0.005778 | 0.778387 | 0.022888 |
